# Vietnam Association of Gastroenterology (VNAGE) consensus on the management of *Helicobacter pylori* infection

**DOI:** 10.3389/fmed.2022.1065045

**Published:** 2023-01-12

**Authors:** Duc Trong Quach, Bang Hong Mai, Mien Kieu Tran, Long Van Dao, Huy Van Tran, Khanh Truong Vu, Khien Van Vu, Ho Thi-Thu Pham, Hoang Huu Bui, Dung Dang-Quy Ho, Dung Tuan Trinh, Vinh Thuy Nguyen, Thai Hong Duong, Tuong Thi-Khanh Tran, Ha Thi-Viet Nguyen, Thinh Tien Nguyen, Thang Duy Nguyen, Long Cong Nguyen, Hang Viet Dao, Ky Doan Thai, Nam Trung Phan, Ly Thanh Le, Cong Hong-Minh Vo, Phat Tan Ho, Tung Lam Nguyen, Quang Dinh Le, Nho Viet Le, Hoan Quoc Phan, Binh Canh Nguyen, Trung Thien Tran, Tu Viet Tran, Long Ta

**Affiliations:** ^1^Department of Internal Medicine, University of Medicine and Pharmacy at Ho Chi Minh City, Ho Chi Minh City, Vietnam; ^2^Nhan Dan Gia Dinh Hospital, Ho Chi Minh City, Vietnam; ^3^108 Military Central Hospital, Hanoi, Vietnam; ^4^Internal Medicine Faculty, Hanoi Medical University, Hanoi, Vietnam; ^5^Hue University of Medicine and Pharmacy, Hue, Vietnam; ^6^Tam Anh Hospital, Hanoi, Vietnam; ^7^Cho Ray Hospital, Ho Chi Minh City, Vietnam; ^8^Department of Internal Medicine, Hanoi National University, Hanoi, Vietnam; ^9^Department of Internal Medicine, Thai Nguyen University of Medicine and Pharmacy, Thai Nguyen, Vietnam; ^10^Department of Internal Medicine, Pham Ngoc Thach University of Medicine, Ho Chi Minh City, Vietnam; ^11^Institute of Gastroenterology and Hepatology, Hanoi, Vietnam; ^12^Bach Mai Hospital, Hanoi, Vietnam; ^13^Department of Internal Medicine, Da Nang University of Medical Technology and Pharmacy, Da Nang, Vietnam; ^14^103 Military Hospital, Hanoi, Vietnam

**Keywords:** consensus, guidelines, *Helicobacter pylori*, Vietnam, diagnosis, eradication, management

## Abstract

*Helicobacter pylori (H. pylori)* infection is prevalent and has a rapidly increasing antibiotic resistance rate in Vietnam. Reinfection is quite common, and gastric carcinoma remains one of the most common malignancies, which is not uncommon to develop after successful eradication. The purpose of this consensus is to provide updated recommendations on the management of *H. pylori* infection in the country. The consensus panel consisted of 32 experts from 14 major universities and institutions in Vietnam who were invited to review the evidence and develop the statements using the Delphi method. The process followed the Grading of Recommendations Assessment, Development, and Evaluation (GRADE) system. The consensus level was defined as ≥80% for agreement on the proposed statements. Due to the limited availability of high-quality local evidence, this consensus was also based on high-quality evidence from international studies, especially those conducted in other populations in the Asia–Pacific region. The panel finally reached a consensus on 27 statements after two voting rounds, which consisted of four sections (1) indications for testing and selection of diagnostic tests (2), treatment regimens, (3) post-treatment confirmation of *H. pylori* status, and (4) reinfection prevention methods and follow-up after eradication. Important issues that require further evidence include studies on third-line regimens, strategies to prevent *H. pylori* reinfection, and post-eradication follow-up for precancerous gastric lesions. We hope this consensus will help guide the current clinical practice in Vietnam and promote multicenter studies in the country and international collaborations.

## Introduction

*Helicobacter pylori* (*H. pylori*) is one of the most common causes of bacterial infections in humans, accounting for over 50% of the world’s population (1). Currently, *H. pylori* gastritis is considered an infectious disease even when it does not cause symptoms or complications (2). Vietnam is one of the countries with the highest rate of *H. pylori* infection and *H. pylori-*induced gastrointestinal diseases in Southeast Asia (3). In Vietnam, the first consensus on managing *H. pylori* infection was developed in 2012 (published in Vietnamese) with recommendations focused on diagnosis and treatment. In the past 10 years, the prevalence of antibiotic-resistant *H. pylori* species has been increasing rapidly, and gastric cancer remains one of the most common and deadly cancers in the country, with the majority of patients being diagnosed at advanced stages (3, 4). Therefore, updating the consensus is an urgent need to guide local clinical practice. This consensus provides recommendations on *H. pylori* diagnosis and treatment as well as reinfection prevention and follow-up strategies after *H. pylori* eradication.

## Methods

### Principles of consensus development

This consensus was developed following the Grading of Recommendations, Assessment, Development, and Evaluation (GRADE) (5). The recommendations in this consensus, prepared by an expert from the Vietnam Association of Gastroenterology, cover issues in four areas: (1) indication and selection of diagnostic tests; (2) *H. pylori* eradication therapies; (3) testing *H. pylori* status after eradication; and (4) reinfection prevention and follow-up after eradication. The drafted statements and supporting evidence were revised by core members and emailed to all panel members 4 weeks before the first virtual meeting. The Delphi method was used to develop consensus. All panel members graded the level of evidence, evaluated the level of agreement and the strength of recommendations for all statements based on the GRADE system, and voted *via* an electronic voting system. They might also suggest additional key references to assess the level of evidence. Regarding the level of consensus, each member will choose one of the following six levels: (1) accept completely (2), accept with some reservation (3), accept with major reservation (4), reject with some reservation (5), reject with major reservation; or (6) reject completely. A statement was approved if the consensus level (calculated based on the total votes at levels 1 and 2) reached ≥80%. The voting members were requested to explain the reasons for the votes that were not at level 1 or 2. Statements that had not reached consensus were revised and discussed in two virtual meetings held on 3 April 2022 and 29 May 2022. Those reaching consensus ≥80% after two voting rounds were used to develop the consensus. The strength of recommendations was rated on two levels: Strong and weak. Statements that received ≥80% of the votes as strong recommendations were considered strong, and the remaining statements were considered weak.

## Results

**FIGURE 1:**
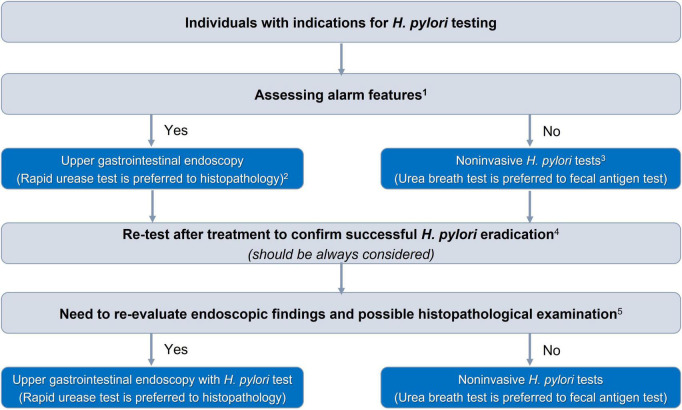
Algorithm for selection of *Helicobacter pylori* diagnostic test. ^1^The alarm features are presented in Table 2. ^2^In patients with acute upper gastrointestinal bleeding, rapid urease test and histopathology can be falsely negative, and *H. pylori* infection should be confirmed after stabilizing gastrointestinal bleeding. ^3^Serum antibody test should not be used. ^4^Patients must stop taking antibiotics or bismuth for at least 4 weeks and PPIs for at least 2 weeks. ^5^Patients diagnosed with gastric ulcers, suspected malignant gastric lesions, or gastric precancerous lesions need further evaluation of their extent and severity.

**FIGURE 2:**
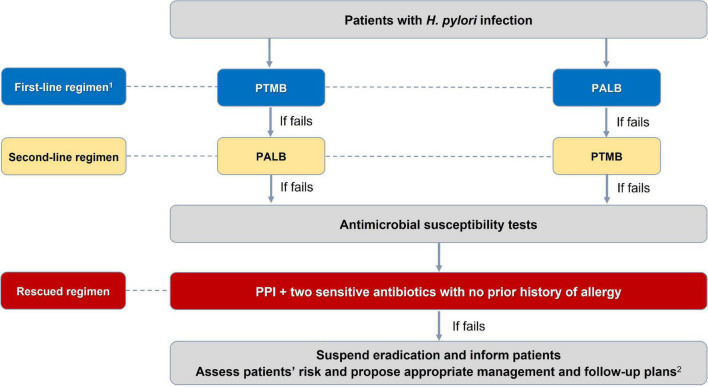
Algorithm for selection of *Helicobacter pylori* eradication regimen. ^1^PTMB is preferred over PALB because of its more stable and higher efficacy. PALB is also not used for people allergic to penicillin. ^2^For patients with *H. pylori*-induced peptic ulcer diseases in whom *H. pylori* have not been successfully eradicated, maintenance anti-secretory therapy is needed. And those with gastric precancerous lesions need appropriate endoscopic follow-up plans to detect early gastric cancer. PTMB: PPI + Tetracycline + Metronidazole + Bismuth; PALB: PPI + Amoxicillin + Levofloxacine + Bismuth. All treatment regimens are in 14 days.

There were 27 consensus statements which are summarized in Table 1. The algorithms for diagnosing and eradicating *H. pylori* infection are also proposed in this consensus (Figures 1, 2).

**TABLE 1:** Summary of recommendations.

Recommendations		Evidence level	Grade of recommendation
**Indication and selection of diagnostic tests**			
Statement 1.	In clinical practice, diagnostic testing for *H. pylori* should be indicated only when eradication therapy is intended.	Low	Strong
Statement 2.	*H. pylori* infection diagnosis is recommended in subjects with the following conditions		
	• Peptic ulcer disease	High	Strong
	• History of peptic ulcer disease but never tested for *H. pylori* infection	High	Strong
	• Uninvestigated dyspepsia	High	Strong
	• Precancerous gastric lesions (i.e., chronic atrophic gastritis, intestinal metaplasia, or gastric dysplasia)	High	Strong
	• After endoscopic resection of early gastric cancer	High	Strong
	• Low-grade MALT lymphoma	High	Strong
	• First-degree relatives diagnosed with gastric cancer	High	Strong
	• Starting and being planned to use long-term non-steroidal anti-inflammatory drugs	High	Conditional
	• Need long-term treatment with low-dose aspirin	High	Conditional
	• Gastroesophageal reflux disease requiring long-term maintenance therapy with proton pump inhibitors	Moderate	Conditional
	• Unexplained iron deficiency anemia	High	Conditional
	• Idiopathic thrombocytopenic purpura	Low	Conditional
	• Individuals who wish to receive *H. pylori* eradication after careful explanation that the treatment is unnecessary.	High	Conditional
Statement 3.	Patients with uninvestigated dyspepsia aged ≥35 years (in females) or ≥40 years (in males) and, or with alarming symptoms should undergo upper gastrointestinal endoscopy. The diagnosis of *H. pylori* infection in these patients should be performed using biopsy-based tests.	Moderate	Strong
Statement 4.	In patients with indications for *H. pylori* infection testing who undergo upper gastrointestinal endoscopy, the rapid urease test is the test of choice.	Moderate	Strong
Statement 5.	Among non-invasive diagnostic tests for *H. pylori* infection, the urea breath test is considered the first choice.	Moderate	Strong
Statement 6.	It is necessary to ensure that patients have not taken any antibiotics or bismuth within 4 weeks or PPIs within 2 weeks before performing diagnostic tests for *H. pylori*.	Moderate	Strong
Statement 7.	In patients with acute upper gastrointestinal bleeding, *H. pylori* testing results using rapid urease test and histopathology can be falsely negative. If these tests are negative, the infection should be confirmed using another highly reliable test after stabilizing gastrointestinal bleeding.	Moderate	Strong
**Treatment regimens for *H. pylori* eradication**			
Statement 8.	A regimen is considered effective and recommended only when its eradication rate is at least 80% (intention to treat). The choice of regimen by this consensus was based on (1) the results from local clinical trials (2), the results of high-quality studies conducted in other countries, and (3) the local experts’ experience.	Moderate	Strong
Statement 9.	The primary resistance rates of clarithromycin and metronidazole are very high. The primary resistance rates of amoxicillin and levofloxacin are on the rise. However, the tetracycline resistance rate is still low and stable.	Moderate	Strong
Statement 10.	Non-adherence to treatment is one of the main causes leading to eradication failure. Spending time counseling and explaining the possible side effects of drugs in eradication regimens can help improve treatment adherence and, consequently, the successful eradication rate.	Moderate	Strong
Statement 11.	Patients should be advised to neither smoke nor drink alcohol during *H. pylori* eradication therapy to avoid reducing eradication efficacy.	Moderate	Conditional
Statement 12.	Good inhibition of acid secretion is one of the critical factors determining the effectiveness of *H. pylori* eradication regimens.	High	Strong
Statement 13.	The optimal duration of all *H. pylori* eradication regimens recommended by this consensus is 14 days.	Moderate	Strong
Statement 14.	Selecting the first-line *H. pylori* eradication regimens		
	A. The first choice regimen is PPI + Tetracycline + Metronidazole + Bismuth (PTMB)	High	Strong
	B. The alternative first-line regimen is PPI + Amoxicilline + Levofloxacin + Bismuth (PALB)	Low	Strong
	C. The Clarithromycin-based triple regimen should not be used due to the high failure rate.	High	Strong
Statement 15.	Selecting the second-line *H. pylori* eradication regimens		
	A. Use the PTMB regimen if it has not been used as a first-line regimen.	High	Strong
	B. Use the PALB regimen if PTMB has been used as a first-line regimen.	Low	Strong
Statement 16.	Selecting salvage regimens after two failed eradication attempts		
	A. The PTMB regimen should be considered if it has not been used.	Moderate	Strong
	B. If the PTMB regimen has been used, antibiotic susceptibility tests should be performed.	Low	Strong
Statement 17.	The rifabutin-based eradication regimen should not be considered due to the complicated situation of antibiotic-resistant tuberculosis in Vietnam.	Very low	Strong
Statement 18.	When patients have well adhered to the appropriate regimens but still do not have successful *H. pylori* eradication, eradication therapy should be suspended. Patients should be informed about appropriate management and follow-up plans until a new and effective eradication regimen is available.	Very low	Conditional
**Testing *H. pylori* status after eradication therapy**			
Statement 19.	Testing for *H. pylori* status should be performed in all patients who have received eradication therapy.	High	Strong
Statement 20.	Upper gastrointestinal endoscopy should be performed in patients with (1) gastric ulcers (2), suspected malignant gastric lesions, or (3) gastric precancerous lesions, which need further evaluation of their extent and severity.	High	Strong
Statement 21.	For individuals who have received *H. pylori* eradication but do not require endoscopic re-evaluation, urea breath tests should be the test of choice to confirm eradication.	High	Strong
**Reinfection prevention and follow-up after *H. pylori* eradication**			
Statement 22.	*H. pylori* is a pathogenic bacterium capable of being transmitted from person to person, especially between members of the same family, through mouth-to-mouth, fecal-oral routes, and contaminated medical devices.	High	Strong
Statement 23.	*H. pylori* reinfection and recrudescence are common in Vietnam.	Low	Strong
Statement 24.	Educating and raising public awareness about the potential sources and transmission routes of *H. pylori* can help reduce the risk of infection in the community.	Moderate	Strong
Statement 25.	In patients who have indications for upper gastrointestinal endoscopy and *H. pylori* biopsy-based tests, the assessment of the presence, severity, and extent of gastric precancerous lesions should always be considered.	Moderate	Strong
Statement 26.	All patients with severe and extensive gastric atrophy or gastric intestinal metaplasia or those with the incomplete subtype of gastric intestinal metaplasia should be followed up endoscopically after successful *H. pylori* eradication.	Moderate	Strong
Statement 27.	All patients with gastric dysplasia detected at mapping biopsies should be endoscopically re-evaluated using image-enhanced endoscopes to establish appropriate surveillance and treatment.	Low	Strong

**TABLE 2:** Alarm features in patients with dyspeptic symptoms.

Alarm features in patients with dyspeptic symptoms
• Progressive dysphagia
• Anemia
• Unintended weight loss
• Evidence of upper gastrointestinal bleeding
• Recurrent or persistent vomiting
• Upper abdominal mass
• New onset dyspepsia in the subjects ≥35 years (in females) or ≥40 years (in males)
• Dyspeptic symptoms that are not responsive to empirical PPI treatment or recur after stopping the treatment for 2–4 weeks.

**I. Indications and selection of diagnostic tests**

**Statement 1.** In clinical practice, diagnostic testing for *H. pylori* should be indicated only when eradication therapy is intended.

*Evidence level: Low*,
*Strength of recommendation: Strong*,
*Consensus level: 100%*,
*Comments: H. pylori* infection is a common bacterial infection, but only approximately 10% of patients with the infection progress to peptic ulcer disease, and 1–3% progress to gastric cancer (6). Vietnam has a very high rate of *H. pylori* infection, with almost two-thirds of the population having positive *H. pylori* serum antibody test results (3, 7). Meanwhile, antibiotic resistance tends to rapidly increase, and there are currently no effective measures to prevent reinfection (4). Consequently, population-based massive screening and eradication are not currently considered in the country.

**Statement 2.** *H. pylori* infection diagnosis is recommended in subjects with the following conditions (Table 1).

Generally, all indications for *H. pylori* diagnostic testing with a high level of evidence from the Maastricht V consensus and the Bangkok consensus on *H. pylori* management in ASEAN countries have been adopted in this consensus (8, 9). These indications include peptic ulcer disease, prior history of peptic ulcer disease but never tested for *H. pylori* infection, uninvestigated dyspepsia, precancerous gastric lesions, after endoscopic resection of early gastric cancer, low-grade MALT lymphoma, having first-degree relatives diagnosed with gastric cancer or starting and being planned to use long-term non-steroidal anti-inflammatory drugs (Table 1).
*Long-term low-dose aspirin therapy*: Peptic ulcer disease occurs in approximately 10% of patients taking low-dose aspirin, most of whom are asymptomatic. Risk factors include age ≥60 years and *H. pylori* infection (10). The risk of peptic ulcer bleeding is almost doubled in patients with *H. pylori* infection, but the number needed to treat to prevent one bleeding case per year is very high, ranging from 100 to 1,000 (11). In patients with a prior history of gastrointestinal bleeding, *H. pylori* eradication markedly reduces recurrent gastrointestinal bleeding and should be considered (12). However, for those who have never had such a history, the costs and benefits of treatment should be carefully considered.
*Gastroesophageal reflux disease requiring long-term maintenance therapy with proton pump inhibitors (PPIs):* Gastric cancer is one of the most common malignancies in Vietnam, and the majority of *H. pylori* infections in Vietnam are virulent strains (3, 13). Long-term treatment with PPIs in patients with *H. pylori* infection may promote corpus-predominant gastritis and atrophy, the two high-risk conditions for gastric cancer development that can be effectively prevented if *H. pylori* is eradicated early (14).
*Unexplained iron deficiency anemia:* A meta-analysis of 16 randomized controlled trials compared the outcomes of two groups of iron-deficient *H. pylori*-infected patients treated with *H. pylori* eradication plus iron supplementation and with iron supplementation alone. The study showed that hemoglobin levels and iron deficiency improved significantly in the former group, especially in patients with moderate or severe anemia (15).
*Idiopathic thrombocytopenic purpura:* A meta-analysis of 6 controlled trials found that *H. pylori* eradication was effective in significantly increasing platelet counts (16). The study, however, was performed on only 241 patients, and the original trials did not clearly describe the randomization process. Therefore, well-designed studies with larger sample sizes are needed.
*Individuals who wish to receive H. pylori eradication after careful explanation that the treatment is unnecessary.* A meta-analysis mainly based on Asian studies reported that *H. pylori* eradication in asymptomatic subjects with *H. pylori* infection reduced the incidence of gastric cancer in regions with high gastric cancer prevalence (17). The best effect is achieved if eradication therapy is performed before mucosal atrophy and gastric metaplasia have occurred (2, 18). Given that gastric cancer is prevalent in Vietnam, individuals with *H. pylori* infection who wish to receive *H. pylori* eradication regimen are justified for the treatment. However, there are still important issues that need further investigation, including the age at which gastric precancerous lesions take off and the long-term adverse consequences on the gut microbiota due to early *H. pylori* eradication in Vietnamese individuals (8).

**Statement 3.** Patients with uninvestigated dyspepsia aged ≥35 years (in females) or ≥40 years (in males) and, or with alarming symptoms should undergo upper gastrointestinal endoscopy. The diagnosis of *H. pylori* infection in these patients should be performed using biopsy-based tests.

*Evidence level: Moderate*,
*Strength of recommendation: Strong*,
*Consensus level: 87.5%*,
Alarm symptoms (Table 2) are not highly sensitive in detecting upper gastrointestinal malignancies (UGIMs) (19, 20). Patient age is an important factor in identifying patients requiring upper gastrointestinal endoscopy. According to the 2011 Asian consensus on the management of dyspepsia, the age threshold to decide upper gastrointestinal endoscopy significantly varies across countries depending on the local prevalence of gastric cancer (21). A recent endoscopic database review of 472,744 Vietnamese patients with upper gastrointestinal symptoms found that there were 2,198 (0.4%) patients with UGIMs. In this study, there were 145 patients with UGIMs whose age was <40 years. Of these patients, 138 (95.2%) were diagnosed with gastric cancer. The age threshold of 35 in women and 40 in men helped to avoid missing UGIMs by 7.1% (95% CI, 5.2–9.4%) and 6.6% (95% CI, 3.3–5.4%), respectively. The age threshold of ≥40 years in women only helped to avoid missing UGIMs by 12.6% (95% CI, 10.1–15.5%) (22). Hormonal factors are suggested to explain why early onset gastric cancer (e.g., age <40 years) is more common in women than in men, but a clear explanation remains unresolved (23).

**Statement 4.** In patients with indications for *H. pylori* infection testing who undergo upper gastrointestinal endoscopy, the rapid urease test is the test of choice.

*Evidence level: Moderate*,
*Strength of recommendation: Strong*,
  *Consensus level: 100%*,
*Comments:* Among the biopsy-based diagnostic tests available in Vietnam, the local rapid urease tests that have been validated should be the test of choice (24, 25). Compared to histopathology, culture, and polymerase chain reaction, these tests are more rapid, much cheaper and have acceptable sensitivity and specificity (except for culture) (26). The other diagnostic methods also require facilities and human resources that are not available in many Vietnamese hospitals.

**Statement 5.** Among non-invasive diagnostic tests for *H. pylori* infection, the urea breath test is considered the first choice.

*Evidence level: Moderate*,
*Strength of recommendation: Strong*,
*Consensus level: 100%*,
*Comments:* One meta-analysis showed that the urea breath test has a sensitivity and specificity of 96 and 93%, respectively (27). Local studies have shown that the urea breath test is as accurate as the rapid urease test (25, 28). There are currently few local data regarding the value of fecal antigen testing. One study in adult patients showed that the test had a sensitivity of 85.7% and a specificity of 71.4% (29). Another study in pediatric patients showed that the test was highly accurate, with a sensitivity and specificity of 96.6 and 94.9%, respectively (30).

**Statement 6.** It is necessary to ensure that patients have not taken any antibiotics or bismuth within 4 weeks or PPIs within 2 weeks before performing diagnostic tests for *H. pylori*.

*Evidence level: Moderate*,
*Strength of recommendation: Strong*,
*Consensus level: 93.7%*,
*Comments:* Diagnostic tests for *H. pylori* infection, which include the urea breath test, rapid urease test, and stool antigen test, can be false negative if patients have recently taken drugs that inhibit the growth of *H. pylori*, such as antibiotics, proton pump inhibitors, and bismuth (31, 32). It should be noted that antibiotics used to treat infections of other organs can also result in false negative tests.

**Statement 7.** In patients with acute upper gastrointestinal bleeding, *H. pylori* testing results using rapid urease test and histopathology can be falsely negative. If these tests are negative, the infection should be confirmed using another highly reliable test after stabilizing gastrointestinal bleeding.

*Evidence level: Moderate*,
*Strength of recommendation: Strong*,
*Consensus level: 81.2%*,
*Comments:* A meta-study showed that the sensitivity of biopsy-based tests, such as rapid urease test, histopathology, and culture, was significantly decreased in patients presenting with acute upper gastrointestinal bleeding (33). A positive result of *H. pylori* serology does not indicate an active infection, and unintended *H. pylori* eradication was reported in about 11% of infected individuals (34). Therefore, the test can be used as a screening tool only. Interestingly, this meta-analysis showed that the accuracy of the urea breath test was still above 90%. Another meta-study showed that repeating diagnostic tests for *H. pylori* at ≥4 weeks after stabilizing gastrointestinal bleeding detected significantly more patients with *H. pylori* infections (35). A local study of 171 patients presenting with peptic ulcer bleeding that used multiple sequential *H. pylori* tests reported that the rate of *H. pylori* infection was 94% (36).

**II. Treatment regimens for *H. pylori* eradication**

**Statement 8.** A regimen is considered effective and recommended only when its eradication rate is at least 80% (intention to treat). The choice of regimen by this consensus was based on: (1) the results from local clinical trials, (2) the results of high-quality studies conducted in other countries, and (3) the local experts’ experience.

*Evidence level: Moderate*,
*Strength of recommendation: Strong*,
*Consensus level: 96.8%*,
*Comments:* The Bangkok consensus states that the ideal eradication rate should be at least 90% (intention to treat) (9). However, local clinical trials in the last 5 years have shown that it is rare for eradication treatment to achieve such a high rate. The required eradication rate of ≥80% according to the WGO Guidelines is probably more appropriate for Vietnam (37). The choice of the regimen was mainly based on the findings of local clinical trials and recent international high-quality trials. The local experts’ experience was considered adjunctive support, especially in situations where the benefits may not outweigh the potential risks, such as the use of a rifabutin-based regimen as tuberculosis is frequent in Vietnam.

**Statement 9.** The primary resistance rates of clarithromycin and metronidazole are very high. The primary resistance rates of amoxicillin and levofloxacin are on the rise. However, the tetracycline resistance rate is still low and stable.

*Evidence level: Moderate*,
*Strength of recommendation: Strong*,
*Consensus level: 100%*,
*Comments:* The results of clinical trials conducted during the last 5 years in Vietnam showed that the primary resistance rates of clarithromycin and metronidazole were very high, up to 34.1 and 69.4%, respectively (4, 38). Recent data also show that the resistance rates of amoxicillin and levofloxacin tend to increase significantly (39, 40). More importantly, there is an emerging increase in multidrug-resistant *H. pylori* strains, which poses a considerable challenge for eradication therapy in clinical practice (41, 42).

**Statement 10.** Non-adherence to treatment is one of the main causes leading to eradication failure. Spending time counseling and explaining the possible side effects of drugs in eradication regimens can help improve treatment adherence and, consequently, the successful eradication rate.

*Evidence level: Moderate*,
*Strength of recommendation: Strong*,
*Consensus level: 96.8%*,
*Comments:* Non-adherence to treatment is one of the leading causes of treatment failure (43, 44). With the same regimen, the eradication rate in non-adherent patients was more than 25% lower than that of adherents (43). Factors affecting the patient’s adherence include complex regimen, long duration of treatment, and lack of instructions and information, especially about the side effects of drugs in the regimen (45). The 2021 WGO guidelines emphasize the use of medication leaflets with the information presented in both text and pictures (37).

**Statement 11.** Patients should be advised to neither smoke nor drink alcohol during *H. pylori* eradication therapy to avoid reducing eradication efficacy.

*Evidence level: Moderate*,
*Strength of recommendation: Weak*,
*Consensus level: 93.7%*,
*Comments:* A meta-analysis showed that smoking while on *H. pylori* eradication therapy could reduce the eradication rate of the same regimen by up to 8.4%. However, the exact mechanism was still not understood (46). Studies on the effect of alcohol consumption on the eradication of *H. pylori* have conflicting results (47, 48). However, it is not uncommon that some Vietnamese people to have a habit of drinking large amounts of alcohol, and being drunk may affect treatment adherence. In particular, when treated with regimens containing metronidazole or tinidazole, patients who drank alcohol during the treatment period are prone to severe side effects such as nausea, vomiting, headache, and flushing (49).

**Statement 12.** Good inhibition of acid secretion is one of the critical factors determining the effectiveness of *H. pylori* eradication regimens.

*Evidence level: High*,
*Strength of recommendation: Strong*,
*Consensus level: 96.8%*,
*Comments:* PPIs are effective in eradicating *H. pylori* and enhancing the effectiveness of antibiotics (50). Recent studies have shown that regimens using esomeprazole and rabeprazole had a better eradication rate of *H. pylori* than first-generation PPIs (i.e., omeprazole, lansoprazole, and pantoprazole) (51, 52). The advantage of rabeprazole and esomeprazole compared to the first-generation PPIs may be due to their higher relative potencies on 24-h gastric pH (53). Indeed, these two PPIs are not concerned by the rapid metabolism due to the hepatic enzyme CYP-2C19, which occurs in some patients due to the genetic polymorphism of the enzyme (54). Dual therapy (PPI plus amoxicillin taken 3–4 times per day) has been reported to be effective in some regions. However, its efficacy needs to be further studied in Vietnam (55). There is emerging evidence that eradication regimens containing potassium-competitive acid secretion inhibitors (PCABs) may be more effective than PPI-based regimens (56, 57). However, PCABs are currently unavailable in Vietnam.

**Statement 13.** The optimal duration of all *H. pylori* eradication regimens recommended by this consensus is 14 days.

*Evidence level: Moderate*,
*Strength of recommendation: Strong*,
*Consensus level: 93.7%*,
*Comments:* For all *H. pylori* eradication regimens, it is recommended to prescribe in 14 days for the best eradication effect (9). Most local trials used 14-day regimens, which showed higher and more stable eradication rates than the shorter regimens (Supplementary material).

**Statement 14.** Selecting the first-line *H. pylori* eradication regimens.

A- The first choice regimen is PPI + Tetracycline + Metronidazole + Bismuth (PTMB).

*Evidence level: High*,
*Strength of recommendation: Strong*,
*Consensus level: 100%*,

B- The alternative first-line regimen is PPI + Amoxicillin + Levofloxacin + Bismuth (PALB).

*Evidence level: Low*,
*Strength of recommendation: Strong*,
*Consensus level: 84.4%*,
*Comments:* Vietnam has very high primary resistance rates to clarithromycin and metronidazole (4). There is strong evidence to use PTMB as a first-line regimen (8). Metronidazole resistance does not affect the eradication results in clinical practice if the antibiotic is used at a dose ≥1,500 mg/day and in combination with bismuth (58). Most clinical trials conducted during the last 5 years in Vietnam have shown that the success rate of the 14-day PTMB regimen was ≥90% (Supplementary material). This regimen is also suitable for patients who are allergic to penicillin. Some local trials also showed that the successful eradication rate of the 14-day levofloxacin-based triple regimen was ≥80%. The eradication rate of this regimen improved when bismuth was added, as reported in previous studies worldwide (59). The recommended doses of antibiotics and PPIs in eradication regimens are summarized in Table 3. Concomitant and sequential regimens are not recommended as first-line regimens, as the eradication rate was lower than that of the two abovementioned regimens, and the evidence level was very low (Supplementary material).

**TABLE 3:** Doses of antibiotics and proton pump inhibitors in eradication regimens.

Antibiotics^1^	Proton pump inhibitors^2^
Amoxicilline 1,000 mg bid	Esomeprazole 40 mg bid
Bismuth 120–240 mg qid	Lanzoprazole 30 mg bid
Clarithromycin 500 mg bid	Omeprazole 40 mg bid
Levofloxacine 500 mg qd	Pantoprazole 40 mg bid
Metronidazole 500 mg bid or tid^3^	Rabeprazole 20 mg bid
Tetracycline 500 mg tid	
Tinidazole 500 mg bid	

The duration of all of the regimens is 14 days.

^1^Antibiotics should be taken with meals. If used four times, one more time should be taken at bedtime.

^2^Proton pump inhibitors should be taken 30 min before breakfast and dinner.

^3^Metronidazole should be used three times per day in a bismuth-based quadruple regimen and two times per day in other regimens.

C- The clarithromycin-based triple regimen should not be used due to the high failure rate.

*Evidence level: High*,
*Strength of recommendation: Strong*,
  *Consensus level: 96.8%*,
*Comments:* In Vietnam, the eradication rate of this regimen dropped from more than 90% in the 2000s to less than 70% in the 2010s (4). It may further decrease due to the increasing prevalence of clarithromycin-resistant strains. Consequently, clarithromycin should not be considered in the eradication regimen unless antibiotic susceptibility tests show it is sensitive.

**Statement 15.** Selecting the second-line *H. pylori* eradication regimens.

A- Use the PTMB regimen if it has not been used as a first-line regimen.

*Evidence level: High*,
*Strength of recommendation: Strong*,
*Consensus level: 100%*,

B- Use the PALB regimen if PTMB has been used as a first-line regimen.

*Evidence level: Low*,
*Strength of recommendation: Strong*,
*Consensus level: 90.6%*,
*Comments:* Several local clinical trials showed that the eradication rate (per protocol) of the 14-day PTMB regimen ranged from 86.7 to 97.6% (Supplementary material). There are few trials on the 14-day PALB regimen. However, the findings suggest that it is also a good option, with an eradication rate of 93.1% in patients whose eradication was unsuccessful with PTMB. Studies on dual regimens have yielded inconsistent results, and further studies are needed (Supplementary material).

**Statement 16.** Selecting salvage regimens after two failed eradication attempts.

A- The PTMB regimen should be considered if it has not been used.

*Evidence level: Moderate*,
*Strength of recommendation: Strong*,
*Consensus level: 100%*,

B- If the PTMB regimen has been used, antibiotic susceptibility tests should be performed.

*Evidence level: Low*,
*Strength of recommendation: Strong*,
*Consensus level: 84.3%*,
  *Comments:* Most of the clinical trials in Vietnam showed that the 14-day PTMB regimen was still effective, with an eradication rate of over 90% if it had not been used. However, one clinical trial reported that the 10-day PTMB regimen has an eradication rate of only 78.9% (Supplementary material). In another open-label non-randomized control trial conducted on patients who had failed eradication twice and PTMB had not been used, two arms of treatment were compared: One with an empiric 14-day PTMB regimen and the other with a tailored 14-day regimen based on antibiotic susceptibility test results. The per-protocol eradication of the former was significantly higher than that of the latter (95.2 vs. 82.8%, *p* < 0.001) (60). The empiric approach was also less expensive.

**Statement 17.** The rifabutin-based eradication regimen should not be considered due to the complicated situation of antibiotic-resistant tuberculosis in Vietnam.

*Evidence level: Very low*,
*Strength of recommendation: Strong*,
*Consensus level: 87.5%*,
*Comments:* Vietnam has a high prevalence of tuberculosis, while the prevalence of *H. pylori* infection is also high, and the rate of secondary antibiotic resistance tends to increase rapidly (3, 4, 7, 61). Therefore, the rifabutin-based regimen should not be prescribed even if this drug is available in Vietnam. This statement has also been mentioned in the WGO guidelines 2021 and the Bangkok Consensus on the management of *H. pylori* in the Southeast Asia Nations 2018 (9, 37).

**Statement 18.** When patients have well adhered to the appropriate regimens but still do not have successful *H. pylori* eradication, eradication therapy should be suspended. Patients should be informed about appropriate management and follow-up plans until a new and effective eradication regimen is available.

*Evidence level: Very low*,
*Strength of recommendation: Weak*,
*Consensus level: 81.2%*,
*Comments:* As multidrug-resistant *H. pylori* strains have rapidly increased, the *H. pylori* infecting patients could be hardly eradicated (41, 60). Further eradication attempts in such patients are unlikely to be successful, potentially increasing secondary resistance and should be avoided. Instead, patients should be informed about appropriate management and follow-up plan until a new and effective regimen is available. For patients with *H. pylori-*induced peptic ulcer diseases in whom *H. pylori* have not been successfully eradicated, maintenance anti-secretory therapy is needed (37). Those with gastric precancerous lesions will need appropriate endoscopic follow-up plans to detect early gastric cancer (62).

**III. Testing *H. pylori* status after eradication**

**Statement 19.** Testing for *H. pylori* status should be performed in all patients who have received eradication therapy.

*Evidence level: High*,
*Strength of recommendation: Strong*,
*Consensus level: 96.8%*,
*Comments:* In patients with *H. pylori* infection who fail eradication, *H. pylori-*induced gastric mucosal injuries may continue to progress, and complications such as peptic ulcer diseases, MALT lymphoma, and gastric carcinoma may eventually develop (2). In real-life practice, most cases of *H. pylori* infection are treated empirically without antibiotic susceptibility testing. Therefore, post-eradication confirmation helps to recognize changes in antibiotic resistance of the bacteria, thereby promptly re-evaluating the evidence and updating recommendations on empiric regimens.

**Statement 20.** Upper gastrointestinal endoscopy should be performed in patients with (1) gastric ulcers, (2) suspected malignant gastric lesions, or (3) gastric precancerous lesions, which need further evaluation of their extent and severity.

*Evidence level: High*,
*Strength of recommendation: Strong*,
*Consensus level: 96.8%*,
*Comments:* Patients with *H. pylori* infection who have gastric ulcers or suspected malignant gastric lesions should have endoscopic re-evaluation after treatment. Approximately 5–10% of gastric ulcers that initially had benign biopsy results were confirmed to be malignant (63). Patients who need endoscopic follow-up include those with precancerous gastric lesions, post-endoscopic resection, early gastric cancer, and gastric adenoma. Rapid urease test has acceptable accuracy, and it is more rapid and much cheaper compared to other endoscopy-based *H. pylori* testing methods.

**Statement 21.** For individuals who have received *H. pylori* eradication but do not require endoscopic re-evaluation, urea breath tests should be the test of choice to confirm eradication.

*Evidence level: High*,
*Strength of recommendation: Strong*,
*Consensus level: 100%*,
*Comments:* The ^13^C urea breath test is preferred to the ^14^C urea breath test for confirming *H. pylori* eradication in such situations. The latter test is cheaper but has the disadvantage of radiation exposure and cannot be used by children and pregnant women (8). The Maastricht V and the Bangkok consensuses recommend using stool antigen test as an alternative test (8, 9). However, there are few studies on the performance of fecal antigen testing in adult patients in Vietnam (29).

**IV. Reinfection prevention and follow-up after eradication**

**Statement 22.** *H. pylori* is a pathogenic bacterium capable of being transmitted from person to person, especially between members of the same family, through mouth-to-mouth, fecal-oral routes, and contaminated medical devices.

*Evidence level: High*,
*Strength of recommendation: Strong*,
*Consensus level: 100%*,
*Comments:* The prevalence of *H. pylori* infection in subjects living in the same Vietnamese family can be as high as 80% (61). The highest prevalence of *H. pylori* infection is in children <12 years of age and in subjects living in multigenerational families. A mother with *H. pylori* infection is the most important risk factor compared to other family members (64). However, other factors may also contribute to the risk of *H. pylori* infection, including living habits, environmental sanitation, and socioeconomic conditions (7, 65). The most common transmission routes are oral-oral, fecal-oral, and transmission among healthcare workers from contaminated equipment (66–68).

**Statement 23.** *H. pylori* reinfection and recrudescence are common in Vietnam.

*Evidence level: Low*,
*Strength of recommendation: Strong*,
*Consensus level: 90.6%*,
*Comments:* The recurrence of *H. pylori* after eradication can be reinfection or recrudescence. *Reinfection* is infection with a new strain of *H. pylori*, which is different from the strain previously eradicated. And *recrudescence* is the relapse of the original *H. pylori* strains temporarily suppressed by eradication therapy (69). Distinguishing between these two situations is quite important in clinical practice, as it relates to the selection of appropriate eradication regimens and to counseling to prevent reinfection. However, it is difficult to assess due to the requirement of culture and molecular typing of the strains. Two independent studies in Vietnam, conducted 15 years apart, showed a recurrence rate of *H. pylori* after 12 months of up to 23% (70, 71). A recent cohort study reported an *H. pylori* recurrence rate of 38.5% at 31-month follow-up (71).

**Statement 24.** Educating and raising public awareness about the potential sources and transmission routes of *H. pylori* can help reduce the risk of infection in the community.

*Evidence level: Moderate*,
*Strength of recommendation: Strong*,
*Consensus level: 93.7%*,
*Comments:* Most *H. pylori* infections occur in young children, whose mothers and direct caregivers are the most important sources of infection (61, 64, 72). Transmission of *H. pylori* to children appears to occur during meals. It is reported that the habit of feeding infants after chewing their food, which is very popular in rural areas in Vietnam, increased the risk of *H. pylori* infection (73). Sharing eating utensils favors oral-oral transmission, but carriage of *H. pylori* by chopsticks is probably very rare (74). The other main risk factors for *H. pylori* infection in children include living in crowded conditions and having a well as the source of home water (75, 76). In adults, *H.* pylori infection may be not associated with crowded living conditions (76). A recent systematic review reported that health professionals, individuals with soil-related occupations, and workers at institutions for the intellectually disabled showed a significantly higher prevalence of *H. pylori* infection than the general population (77).

**Statement 25.** In patients who have indications for upper gastrointestinal endoscopy and *H. pylori* biopsy-based tests, the assessment of the presence, severity, and extent of gastric precancerous lesions should always be considered.

*Evidence level: Moderate*,
*Strength of recommendation: Strong*,
*Consensus level: 96.8%*,
*Comments:* Precancerous gastric lesions are very frequent in Vietnamese patients presenting with dyspepsia (78, 79). Assessing the presence, severity, and extent of precancerous gastric lesions at the index endoscopy is crucial to stratify the risk of gastric cancer for an appropriate surveillance strategy. Image-enhanced endoscopy should be used as it provides a more accurate assessment of precancerous gastric lesions compared to white light endoscopy (80). For regions with limited resources, endoscopic assessment of gastric atrophy according to the Kimura–Takemoto classification can help identify subjects at high risk of gastric cancer who need to be followed up after *H. pylori* eradication (2, 80, 81). A recent study in Japan showed that the predictive value for gastric cancer development of this endoscopic classification was not inferior to well-validated histopathological classifications such as the operative link for gastritis assessment (OLGA) and operative link for gastric intestinal metaplasia (OLGIM) assessment (82).

**Statement 26.** All patients with severe and extensive gastric atrophy or gastric intestinal metaplasia or those with the incomplete subtype of gastric intestinal metaplasia should be followed up endoscopically after successful *H. pylori* eradication.

*Evidence level: Moderate*,
*Strength of recommendation: Strong*,
*Consensus level: 100%*,
*Comments:* The risk of gastric cancer development depends on the severity and extent of precancerous gastric lesions (2, 81–83). Gastric cancer may develop in some patients after successful eradication, especially in those who have already had extensive, severe precancerous gastric lesions or incomplete subtype of gastric intestinal metaplasia prior to eradication (2, 84). The time interval for endoscopically following up patients with precancerous lesions after eradication has been addressed in international guidelines, ranging from 1 to 3 years depending on other risk factors, such as a family history of gastric cancer, *H. pylori* infection status, and gastric intestinal metaplasia subtype (62, 85). Local evidence is limited, and further studies in Vietnamese are awaited.

**Statement 27.** All patients with gastric dysplasia detected at mapping biopsies should be endoscopically re-evaluated using image-enhanced endoscopes to establish appropriate surveillance and treatment.

*Evidence level: Low*,
*Strength of recommendation: Strong*,
*Consensus level: 87.5%*,
*Comments:* Approximately 2.5% of cases with low-grade gastric dysplasia were identified based on mapping biopsies without any endoscopically suspected findings. Some may present with gastric dysplasia in multiple biopsy sites in the same patient (86). Image-enhanced endoscopy is recommended for endoscopic re-evaluation of these patients due to its better ability to identify dysplastic lesions compared to white light endoscopy (62, 85). Dysplastic lesions that are endoscopically detected should be resected for accurate histopathology (62, 85). In cases where dysplastic lesions are not endoscopically detected, patients should be re-evaluated endoscopically after 6 months (for high-grade gastric dysplasia) or 12 months (for low-grade gastric dysplasia) (62, 85).

## Conclusion

*H. pylori* is a common infection in Vietnam with a rapidly increasing antibiotic resistance rate. Gastric cancer is one of the most common malignancies in the country and can occur even after successful *H. pylori* eradication. This consensus provides recommendations on managing *H. pylori* infection and the post-eradicated follow-up strategies in Vietnam. Important clinical issues that require additional local evidence for future recommendations include effective third-line regimens, reinfection prevention methods, and specific post-eradicated follow-up plans for patients with gastric precancerous lesions. We hope that this consensus will be a helpful tool to guide clinical practice in Vietnam and promote international research collaborations.

## Author contributions

DQ drafted all the statements and prepared the supporting literature and further revisions were done by BM, LT, MT, LD, KTV, and KVV. BM, LT, MT, LD, KTV, and DQ edited the revised statements. DQ drafted, critically revised, and submitted the manuscript. All authors approved the final version of the manuscript.
