# Supplementary material for: Vietnam Association of Gastroenterology (VNAGE) consensus on the management of Helicobacter pylori infection
Source: Front Med (Lausanne). 2023 Jan 12;9:1065045. doi: 10.3389/fmed.2022.1065045 (PMC9878302; doi:10.3389/fmed.2022.1065045)
Supplement: Supplementary file 1 [file Data_Sheet_1.DOCX]

**SUPPLEMENT 1. *H. PYLORI* ERADICATION RATES IN CLINICAL TRIALS IN VIETNAM**

1. ***H. pylori* eradication rates of first-line regimens in Vietnam**

| Authors | Year | Region | N | Loss of Follow up | Regimen | Duration | Eradication Rate (Intention-to-treat) | Eradication Rate (Per protocol) | Adverse Effect |
| --- | --- | --- | --- | --- | --- | --- | --- | --- | --- |
| Dang NQ Hue et al ^1^ | 2016 | Central | 122 | 12.3% | EBMT | 10 | 79.5% (71.3-86.3) | 90.6% (83.5-95.4) | 81.9% (75.2-87.5) |
| Bui C Nam et al ^2^ | 2016 | North | 306 | 0% | PBMT | 14 | 88.6% (84.4-91.9) | 88.6% (84.4-91.9) | 36.6% (30.3-41.3) |
| Tran TK Tuong et al ^3^ | 2017 | South | 115 | 6.1% | RBMT | 14 | 92.1% (85.6-96.3 | 98.1% (93.5-99.8) | 80.6% (74.4-85.9) |
| Tran V Huy et al ^4^ | 2019 | Central | 60 | 16.7% | RBMT | 14 | 75% (62.1-85.3) | 90% (78.2-96.7) | 48.3% (35.2-61.6) |
| Tran TK Tuong et al ^5^ | 2020 | South | 252 | 12.3% | RBMT | 14 | 84.1% (80.3 -94.2) | 95.9% (88.5- 9.2) | 73.3% (66.9-79) |
| Thai TH Nhung et al ^6^ | 2020 | South | 40 | 10% | RBTTi | 14 | 95% (83.1-99.4) | 97.2% (85.5-99.9) | 75% (58.8-87.3) |
| Khuc T Trang et al ^7^ | 2021 | North | 233 | 6.8% | EBTTi | 14 | 85.3% (80.8-89.8) | 91.7% (88-95.4) | 80.3% (74.6-85.2) |
| Dao H Ngoi et al ^8^ | 2010 | South | 175 | 22.8% | OAL | 14 | 68% (60.5-74.8) | 88.2 % (81.5-93.1) | 37% (28.9-45.8) |
| Tran TK Tuong ^9^ | 2020 | South | 239 | 3.3% | RAL | 14 | 77.4% (73.6- 83.5) | 80.1% (76.5-88.3) | 36.4% (30.1-42.9) |
| Nguyen TN Doan ^10^ | 2018 | South | 89 | 2.2% | PALB | 14 | 91% (83.1-96%) | 93.1% (85.5-97.4) | 59.4% (46.4-71.5) |
| Tran TK Tuong ^9^ | 2020 | South | 89 | 7.8% | RALB | 14 | 91% (83-96) | 92.7% (84.8-97.2) | 38.2% (28.1-49.1) |
| Vinh Khanh et al ^11^ | 2011 | Central | 81 | 23.4% | RACM | 5 | 67.9% (56.6-77.8) | 88.7% (78.1-95.3) | 17.7% (9.2-29.5) |
| Truong V Lam et al ^12^ | 2014 | South | 53 | 0% | EACM | 10 | 88.7% (76.9-95.7) | 88.7% (76.9-95.7) | 39.6% (26.5-54) |
| Thai T Hoai et al ^13^ | 2016 | Central | 83 | 0% | RACM | 14 | 83.1% (73.3-90.5) | 83.1% (73.3-90.5) | - |
| Nguyen TT Van et al ^14^ | 2017 | Central | 76 | 9.2% | RALTi | 10 | 82.9% (72.5-90.6) | 91.3% (82-96.7) | 34.8% (23.7-47.2) |
| Bui H Hoang ^15^ | 2011 | South | 40 | 10% | PA-PCTi | 10 | 77.5% (61.5-89.2) | 86.1% (70.5-95.3) | 77.8% (60.8-89.9) |
| Tran V Huy et al ^16^ | 2013 | Central | 42 | 16.7% | RA-RCTi | 10 | 73.8% (57.9-86.1) | 88.6% (73.2-96.8) | 25% (12.1-42.2) |
| Truong V Lam et al ^17^ | 2013 | South | 68 | 14.7% | RA-RCTi | 10 | 73.5% (61.4-83.5) | 86.2% (74.6-93.8) | 29.3% (18.1-42.7) |
| Truong V Lam et al ^12^ | 2014 | South | 56 | 0% | EA-RCTi | 10 | 83.9% (71.7-92.4) | 83.9% (71.7-92.4) | 31.1% (20.3-45.9) |
| Nguyen PH Ngoc et al ^18^ | 2017 | Central | 102 | 9.8% | RA-RLTi | 10 | 73.5% (63.8-81.1) | 81.5% (72.1-88.8) | 65.2% (54.6-74.8) |
| Hoang T Thang et al ^19^ | 2018 | Central | 73 | 13.7% | EA-EACTi | 14 | 78.1% (66.9-86.9 | 90.5% (80.4-96.4) | 47.9% (36.1-60) |
| Pham N Doanh et al ^20^ | 2019 | Central | 116 | 6% | RA-RLTi | 10 | 81.9% (73.7-88.4) | 87.2% (79.4-92.8) | 33.9% (25.2-43.6) |
| ***Abbreviations:*** *E: Esomeprazole, O: Omeprazole, P: Pantoprazole, R: Rabeprazole, A: Amoxicillin, B: Bismuth, C: Clarithromycin, L: Levofloxacine, M: Metronidazole, Ti: Tinidazole.* | | | | | | | | | |

1. ***H. pylori* eradication rates of second-line regimens in Vietnam**

| Authors | Year | Region | N | Loss of Follow up | Regimen | Duration | Eradication Rate (ITT) | Eradication Rate (PP) | Adverse Effect |
| --- | --- | --- | --- | --- | --- | --- | --- | --- | --- |
| Nguyen T Vinh ^21^ | 2011 | North | 45 | 0% | EBMT | 14 | 86.7% (73.2-94.9) | 86.7% (73.2-94.9) | 46.7% (31.7-62.1) |
| Dang NQ Hue et al ^1^ | 2016 | South | 24 | 0% | EBMT | 10 | 91.7% (73-98.9) | 91.7% (73-98.9) | 81.9% (75.2-87.5) |
| Tran TK Tuong et al ^3^ | 2017 | South | 43 | 2.3% | RBMT | 14 | 95.3% (84.2-99.4) | 97.6% (87.4-99.9) | 80.6% (74.4-85.9) |
| Bui Q Di et al ^22^ | 2012 | Central | 101 | 0% | RAL | 10 | 83.2% (74.4-89.9) | 83.2% (74.4-89.9) | 7.9% (3.5-15.0) |
| Bui C Nam et al ^2^ | 2016 | North | 80 | 0% | PAL | 10 | 60% (48.4-70.8) | 60% (48.4-70.8) | 36.6% (30.3-41.3) |
| Tran TK Tuong ^23^ | 2019 | South | 113 | 3.5% | RALB | 14 | 90.3% (83.2-95) | 93.1% (86.4-97.2) | 44.2% (34.9-53.9) |
| Tran TK Tuong ^24^ | 2019 | South | 89 | 5.6% | RA | 14 | 86.5% (77.6-92.8) | 91.7% (83.6-96.6) | 11.24% (5.5-19.7) |
| Bui H Hoang et al ^25^ | 2020 | South | 27 | 3.7% | RA | 14 | 25.9% (11.1-46.3) | 26.9% (11.6-47.8) | NA |
| ***Abbreviations:*** *E: Esomeprazole, O: Omeprazole, P: Pantoprazole, R: Rabeprazole, A: Amoxicillin, B: Bismuth, C: Clarithromycin, L: Levofloxacine, M: Metronidazole, Ti: Tinidazole, NA:non applicable.* | | | | | | | | | |

1. ***H. pylori* eradication rates of third-line regimens in Vietnam**

| Authors | Year | Region | N | Loss of Follow up | Regimen | Duration | Eradication Rate (ITT) | Eradication Rate (PP) | Adverse Effect |
| --- | --- | --- | --- | --- | --- | --- | --- | --- | --- |
| Dang NQ Hue et al ^1^ | 2016 | South | 20 | 5% | EBMT | 10 | 75% (50.9-91.3) | 78.9% (54.4-93.9) | 81.9% (75.2-87.5) |
| Tran TK Tuong et al ^3^ | 2017 | South | 38 | 13.1% | RBMT | 14 | 81.5% (65.6-91.2) | 93.9% (79.8-99.3) | 80.6% (74.4-85.9) |
| Bui H Hoang et al ^26^ | 2017 | South | 77 | 0% | EAL | 14 | 81.8% (71.4-89.7) | 81.8% (71.4-89.7 | NA |
| Bui H Hoang et al ^26^ | 2017 | South | 69 | 0% | EAT | 14 | 89.8% (80.2-95.8) | 89.8% (80.2-95.8) | NA |
| Bui H Hoang et al ^26^ | 2017 | South | 189 | 0% | EBMT | 14 | 95.2% (91.1-97.8) | 95.2% (91.1-97.8) | NA |
| Ho T Phat et al ^27^ | 2018 | South | 97 | 1% | EBMT | 14 | 94.5% (88.4-98.3) | 95.8% (89.7-98.8) | 77% (67.4-85.0) |
| ***Abbreviations:*** *E: Esomeprazole, O: Omeprazole, P: Pantoprazole, R: Rabeprazole, A: Amoxicillin, B: Bismuth, C: Clarithromycin, L: Levofloxacine, M: Metronidazole, Ti: Tinidazole, NA:non applicable.* | | | | | | | | | |

***References (Fulltext in Vietnamese)***

[1] Dang NQH TV, Nguyen TH. Chronic Helicobacter pylori gastritis: The eradication efficacy of the Bismuth-containing quadruple regimen (EBMT). *Journal of Medicine and Pharmacy*. 2016; **32**: 148-56.

[2] Bui CN NC, Nguyen TTH, Nguyen TC, Nguyen XT. Efficacy of Helicobacter pylori eradication therapy with PCA, PTMB, PLA. *Vietnamese Journal of Gastroenterology*. 2016; **IX**: 2851-54.

[3] Tran TKT VQ. The efficacy of the Bismuth-containing quadruple regimen in Helicobacter pylori infection treatment. *Journal of Medicine and Pharmacy*. 2017; **7**: 29-34.

[4] Tran VH. Efficacy of modified bismuth quadruple therapy (RBMA) as first-line therapy for eradication of Helicobacter pylori in patients with chronic gastritis. *Journal of Medicine and Pharmacy*. 2019; **9**.

[5] Tran TKT HQ, Tong NDH, Nguyen TAD, Ngo TTQ. Bismuth quadruple therapy versus Levofloxacin triple therapy for first-line Helicobacter pylori eradication treatment: a multicenter study. *Medical science*. 2020; **24**.

[6] Thai THN HH. The clinical characteristics, endoscopic findings and the efficacy of the Bismuth-containing quadruple regimen in treatment of patients with Helicobacter pylori gastroduodenal inflammatory and ulcer at can tho

hospital of university of medicine and pharmacy. *Journal of Medicine and Pharmacy*. 2020; **3**: 7-12.

[7] Khuc TT NC, Vu TK. The efficacy of Bismuth-containing quadruple regimen in duodenal ulcer bleeding patients with Helicobacter pylori. *Vietnamese Journal of Gastroenterology*. 2021; **IX**: 3842-48.

[8] Dao HN NC, Nguyen TTT, Nguyen HT, Le TTT, Nguyen TTT, To VQ, Bui HH. The effectiveness of Omeprazole + Amoxicillin + Levofloxacin regimen compared with Omeprazole + Amoxicillin + Clarithromycin regimen in eradication of Helicobacter pylori infection in patients with gastritis and peptic ulcer disease. *Hochiminh City Journal of Medicine*. 2010; **14** 184-9.

[9] Tran TKT. The Eradication Rate of Levofloxacin containing Quadruple therapy for the First-Line treatment of Helicobacter Pylori. *Biomedical Journal of Scientific & Technical Research*. 2020; **Volume 27**: 20494-96.

[10] Nguyen TND. Efficacy of Levofloxacin Bismuth quadruple-based therapy for Helicobacter pylori. *Vietnamese Journal of Gastroenterology*. 2018; **IX**: 3294-99.

[11] Vinh K PN, Tran VH. Study on efficacy of quadruple therapy with RACM in patients with positive Helicobacter pylori duodenal gastric ulcer. *Journal of Medicine and Pharmacy*. 2011; **5**: 88-99.

[12] Truong VL MT, Nguyen MN, Vo XL. The effectiveness of concomitant therapy and sequential therapy in Helicobacter pylori eradication in An Giang center general hospital. *Proceedings of the Scientific Conference of An Giang Hospital*. 2014; **October**: 58-71.

[13] Thai TH TV. Clinical, endoscopic and pathological responses after eradication with RACM regimen in patients of Helicobacter pylori-related chronic gastritis. *Journal of Medicine and Pharmacy*. 2016; **32**: 12-9.

[14] Nguyen TTV. Efficacy of Rabeprazole - Amoxicillin - Levofloxacin – Tinidazole regimen in duodenal ulcer patients with positive Helicobacter pylori. . *Doctor's thesis in Internal Medicine Hue University of Medicine and Pharmacy*. 2017.

[15] Bui HH. The effect of sequential therapy in eradication of Helicobacter pylori in patients with gastritis and peptic ulcer disease. *Hochiminh City Journal of Medicine*. 2011; **15**: 303-7.

[16] Tran VH LM. Efficacy of sequential therapy (RA-RCT) in Helicobacter pylori eradication in patients with chronic gastritis. *Journal of Medicine and Pharmacy*. 2013; **4**: 24-30.

[17] Truong VL MT, Nguyen MN, Nguyen KL, Nguyen NR. Sequential therapy in comparison with the standard triple therapy for eradicating Helicobacter pylori infection: A randomized controlled study. *Hochiminh City Journal of Medicine*. 2013; **17**.

[18] Nguyen PHN. Efficacy of sequential therapy with Levofloxacin in gastritis patients with positive Helicobacter pylori. *Master's thesis in Internal Medicine Hue University of Medicine and Pharmacy*. 2017.

[19] Hoang TT PP. Study the efficacy of 14 days hybrid therapy of EA - EACT on patients with positive Helicobacter pylori duodenal ulcer. . *Vietnamese Journal of Gastroenterology*. 2018; **IX**: 3239-45.

[20] Pham ND. Assessment of Clarithromycin resistant strain of Helicobacter pylori by PCR-RFLP and the efficacy of Levofloxacin-based sequential therapy in gastritis patients. *Doctor's thesis in Internal Medicine Hue University of Medicine and Pharmacy*. 2019.

[21] Nguyen TV. A study to assess Helicobacter pylori eradication rate of EAC and EBTM regiments in clinical practice. *Journal of Practical Medicine*. 2011; **760**: 23-6.

[22] Bui QD HT. A study to assess the efficacy of triple therapy with Levofloxacin-based regime in the eradication of Helicobacter pylori. *Vietnamese Journal of Gastroenterology*. 2012; **VII**: 1730-4.

[23] Tran TKT. Efficacy of Levofloxacin-based quadruple therapy in positive Helicobacter pylori patients after failure of standard triple therapies. . *Summary of abstracts in 25^th^ National Vietnamese Gastroenterology Conference*. 2019; **A03**: 11.

[24] Tran TKT. Efficacy and safety of High‐dose Dual therapy for eradication of Helicobacter pylori after failure of standard Clarithromycin-triple therapy. *Hochiminh City Journal of Medicine*. 2019; **23**: 254-8.

[25] Bui HH VH, Trinh TTT, Nguyen TBD. Efficacy of Helicobacter pylori eradication therapy with HDDT therapy in positive Helicoacter pylori patients with peptic diseases. *Summary of abstracts in 26^th^ National Vietnamese Gastroenterology Conference*. 2020; **AB 015**: 41.

[26] Bui HH LT, Luong BA, Do TTT, . Application of antimicrobial susceptibility testing and CYP2C19 polymorphism in Helicobacter pylori eradication in patients after treatment failure. *Hochiminh City Journal of Medicine*. 2017; **21**: 120-9.

[27] Ho TP TN, Tong PH, Bui HH,Vu TKT, Ma PN, Tran TDT, Nguyen VA, Nguyen HC, Tran TKN, Le TNH, Nguyen THD. Investigating the efficacy of Bismuth quadruple therapy and CYP2C19 polymorphism’s influence on eradication of Helicobacter pylori after previous treatment failure. *Hochiminh City Journal of Medicine*. 2018; **22**: 98-104.
